# The Intellectual Disability Risk Gene *Kdm5b* Regulates Long-Term Memory Consolidation in the Hippocampus

**DOI:** 10.1523/JNEUROSCI.1544-23.2024

**Published:** 2024-04-04

**Authors:** Leticia Pérez-Sisqués, Shail U. Bhatt, Rugile Matuleviciute, Talia E. Gileadi, Eniko Kramar, Andrew Graham, Franklin G. Garcia, Ashley Keiser, Dina P. Matheos, James A. Cain, Alan M. Pittman, Laura C. Andreae, Cathy Fernandes, Marcelo A. Wood, K. Peter Giese, M. Albert Basson

**Affiliations:** ^1^Centre for Craniofacial and Regenerative Biology, Guy’s Hospital, King’s College London, London SE1 9RT, United Kingdom; ^2^MRC Centre for Neurodevelopmental Disorders, Institute of Psychiatry, Psychology and Neuroscience, King’s College London, London SE1 1UL, United Kingdom; ^3^Department of Neurobiology and Behavior, School of Biological Sciences, University of California Irvine, Irvine, California, California 92697; ^4^St. George’s University of London, London SW17 0RE, United Kingdom; ^5^Social, Genetic & Developmental Psychiatry Centre, Institute of Psychiatry, Psychology and Neuroscience, King’s College London, London SE5 8AB, United Kingdom; ^6^Department of Basic and Clinical Neuroscience, Institute of Psychiatry, Psychology and Neuroscience, King’s College London, Maurice Wohl Clinical Neuroscience Institute, London SE5 9RT, United Kingdom; ^7^Department of Clinical and Biomedical Sciences, University of Exeter Medical School, Hatherly Laboratories, Exeter EX4 4PS, United Kingdom

**Keywords:** chromatin, hippocampus, histone lysine demethylase, KDM5B, learning, memory, mouse

## Abstract

The histone lysine demethylase KDM5B is implicated in recessive intellectual disability disorders, and heterozygous, protein-truncating variants in *KDM5B* are associated with reduced cognitive function in the population. The KDM5 family of lysine demethylases has developmental and homeostatic functions in the brain, some of which appear to be independent of lysine demethylase activity. To determine the functions of KDM5B in hippocampus-dependent learning and memory, we first studied male and female mice homozygous for a *Kdm5b*^*ΔARID*^ allele that lacks demethylase activity. *Kdm5b*^*ΔARID/ΔARID*^ mice exhibited hyperactivity and long-term memory deficits in hippocampus-dependent learning tasks. The expression of immediate early, activity-dependent genes was downregulated in these mice and hyperactivated upon a learning stimulus compared with wild-type (WT) mice. A number of other learning-associated genes were also significantly dysregulated in the *Kdm5b*^*ΔARID/ΔARID*^ hippocampus. Next, we knocked down *Kdm5b* specifically in the adult, WT mouse hippocampus with shRNA. *Kdm5b* knockdown resulted in spontaneous seizures, hyperactivity, and hippocampus-dependent long-term memory and long-term potentiation deficits. These findings identify KDM5B as a critical regulator of gene expression and synaptic plasticity in the adult hippocampus and suggest that at least some of the cognitive phenotypes associated with *KDM5B* gene variants are caused by direct effects on memory consolidation mechanisms.

## Significance Statement

The histone lysine demethylase KDM5B has been implicated in cognitive performance and intellectual disability conditions in the human population. In the present manuscript, we show that mice expressing a demethylase-deficient KDM5B and mice with a specific knockdown of KDM5B in the adult hippocampus exhibit hippocampus-dependent learning and memory phenotypes. Molecular analyses suggest a key role for KDM5B in regulating the dynamic expression of activity-regulated genes during memory consolidation. Deficits in long-term potentiation are present in mice with KDM5B knockdown. Together, these findings provide the first evidence for a direct function for KDM5B in memory consolidation in the hippocampus.

## Introduction

Genome sequencing studies have implicated genes encoding chromatin modifying factors in intellectual disability and autism spectrum disorders (ID and ASDs). These include the gene *KDM5B* (lysine demethylase 5B), recessive mutations of which are linked to a rare ID syndrome (Faundes et al., 2018; Martin et al., 2018). Intriguingly, *KDM5B* is tolerant to loss-of-function mutations, and a significant proportion of such heterozygous *KDM5B* variants are incompletely penetrant and inherited (T. Wang et al., 2022; Zhou et al., 2022). A recent study has reported that rare protein-truncating variants in *KDM5B* are associated with large effects on cognitive function in the population (C. Y. Chen et al., 2023). Specifically, protein-truncating variants in *KDM5B* negatively correlated with educational attainment, reaction time, and verbal–numerical reasoning. This study also reported an association of protein-truncating variants of *KDM5B* with neurodevelopmental and psychiatric disorders and epilepsy. The same study found initial evidence that a heterozygous and homozygous deletion of *Kdm5b* exon 7 in mice was associated with dose-dependent cognitive deficits and increased anxiety (C. Y. Chen et al., 2023). Previous studies have reported a significant embryonic or perinatal lethality of homozygous *Kdm5b* loss-of-function mutations in mice. The deletion of exon 1 was associated with lethality by embryonic day 7.5 (Catchpole et al., 2011), whereas either exon 6 (Albert et al., 2013) or exon 7 (Martin et al., 2018) deletion was associated with 36–44% postnatal viability, suggesting that the deletion of exons 6 or 7 produces hypomorphic alleles with partially penetrant effects.

KDM5B, together with KDM5A and KDM5C, are the only histone lysine demethylases known to demethylate trimethylated lysine 4 on histone 3 (H3K4me3), a post-translational histone modification typically present at active gene promoters. H3K4me3 can recruit components of transcriptional initiator (Vermeulen and Timmers, 2010; Lauberth et al., 2013) and integrator complexes (H. Wang et al., 2023). The latter is associated with the regulation of transcriptional output by stimulating transcriptional elongation (H. Wang et al., 2023). As KDM5B specifically removes H3K4me3 to counteract this process, the lack of KDM5B demethylase activity is predicted to lead to transcriptional derepression of direct target genes. H3K4me3 is also enriched at nonmethylated CpG island promoters, where it is thought to counteract DNA methylation (Hughes et al., 2020).

The *KDM5A-D* genes in mammals are paralogues of the ancestral *kdm5* gene in *Drosophila*. *kdm5* mutation is also associated with lethality (Gildea et al., 2000). *Drosophila kdm5* can regulate gene expression with multiple mechanisms, including a mechanism that requires the PHD domain of the protein, and appears to function independently from the demethylase activity of the protein (Liu and Secombe, 2015). Indeed, *Drosophila* *kdm5* and mammalian KDM5 proteins interact with other chromatin factors and complexes that function as transcriptional repressors, including components of Sin3/HDAC1 and NuRD complexes (Yheskel et al., 2023). Thus, some KDM5B-mediated transcriptional repression may be mediated by demethylase-independent mechanisms. Flies with mutations in the demethylase domains of the protein survive, consistent with demethylase-independent functions, but exhibit specific learning phenotypes (Zamurrad et al., 2018; Belalcazar et al., 2021).

Mutations in the lysine methyltransferases responsible for H3K4me3 (KMT2A and KMT2B), are also associated with autosomal dominant ID syndromes (W. D. Jones et al., 2012; Faundes et al., 2018). Animal experiments have shown that a learning stimulus results in a rapid and transient increase in H3K4me3 levels in the hippocampus, similar to immediate early gene induction (Gupta et al., 2010). Consistent with a functional role for H3K4me3, the lysine methyltransferases, KMT2A and KMT2B are necessary for normal hippocampus-dependent learning and memory in mice (Kerimoglu et al., 2013, 2017).

Together, these studies suggest that dysregulation of H3K4me3, either by loss of function of methyltransferases or demethylases, can result in ID. The conditional deletion of *Kmt2a* and *Kmt2b* genes in postmitotic neurons in the mouse is sufficient to cause learning and memory phenotypes, suggesting that defects in the H3K4me3 machinery directly impact memory consolidation mechanisms in the hippocampus (Kerimoglu et al., 2013, 2017). Thus, to understand how KDM5B loss of function affects learning and memory, we set out to determine if KDM5B has a direct function in memory consolidation, as opposed to having exclusively developmental functions. We focused on the hippocampus as the function of this brain region in learning and memory is well established, and hippocampus-dependent memory tasks and electrophysiological correlates such as long-term potentiation (LTP) are robust in mice.

## Materials and Methods

### Animals

Experiments with mice carrying the *Kdm5b^tm1Jtpu^*, referred to as the *Kdm5b^ΔARID^* allele (Catchpole et al., 2011), were performed at King's College London. *Kdm5b^ΔARID/ΔARID^* males on a C57BL/6J background were crossed with 129S2/Sv females to produce heterozygous F1 mice that were then intercrossed to produce wild-type (WT) and homozygous *Kdm5b^Δ/Δ^* F2 mice for experiments. Experimental cohorts of mice were derived from 5 (studies with naive mice) to 15 (for behavioral analyses) litters. Animals were housed in ventilated cages (37 × 20 × 16 cm) with *ad libitum* access to water and food (LabDiet PicoLab rodent-irradiated diet, #5R53) and kept at 19–22°C and 40–60% humidity, under a 12:12 h light/dark cycle. The cages contained bedding (Lignocel wood fiber) and nesting. A maximum of five animals were housed in the same cage. All animal procedures were approved by the King's College London AWERB and the UK Home Office.

### Genotyping of mice

Genotyping was performed by extracting genomic DNA from ear notches as previously described (Hurley et al., 2021). The following primer pairs to detect the WT and the mutant alleles were used: WT forward 5′-CCTTAGACGCAGACAGCACA-3′, WT reverse 5′-CGTGTTTGGGCCTAAATGTC-3′, Kdm5b-ΔARID forward 5′-TGCTCCTGCCGAGAAAGTATCC-3′, and Kdm5b-ΔARID reverse 5′-CCACCCCCCAGAATAGAATGA-3′. Thermal cycles for the genotyping reactions were as follows: 95°C, 2 min; 35× (95°C, 15 s; 64°C, 15 s; 72°C, 15 s); 72°C, 12 min.

### *Kdm5b* knockdown

Adult (8–12 week old) female C57BL/6J mice received 1 µl (1 × 10^10^ viral particles) of either AAV1-CMV-GFP-U6-mKdm5b-shRNA (Vector Biolabs #shAAV-262769) or control AAV1-CMV-GFP-U6-scrambled shRNA (#7040) in both dorsal hippocampi as described (Kwapis et al., 2018). These experiments were approved by the UCI IACUC and KCL AWERB and UK Home office.

### Western blot

**Figure 1.**
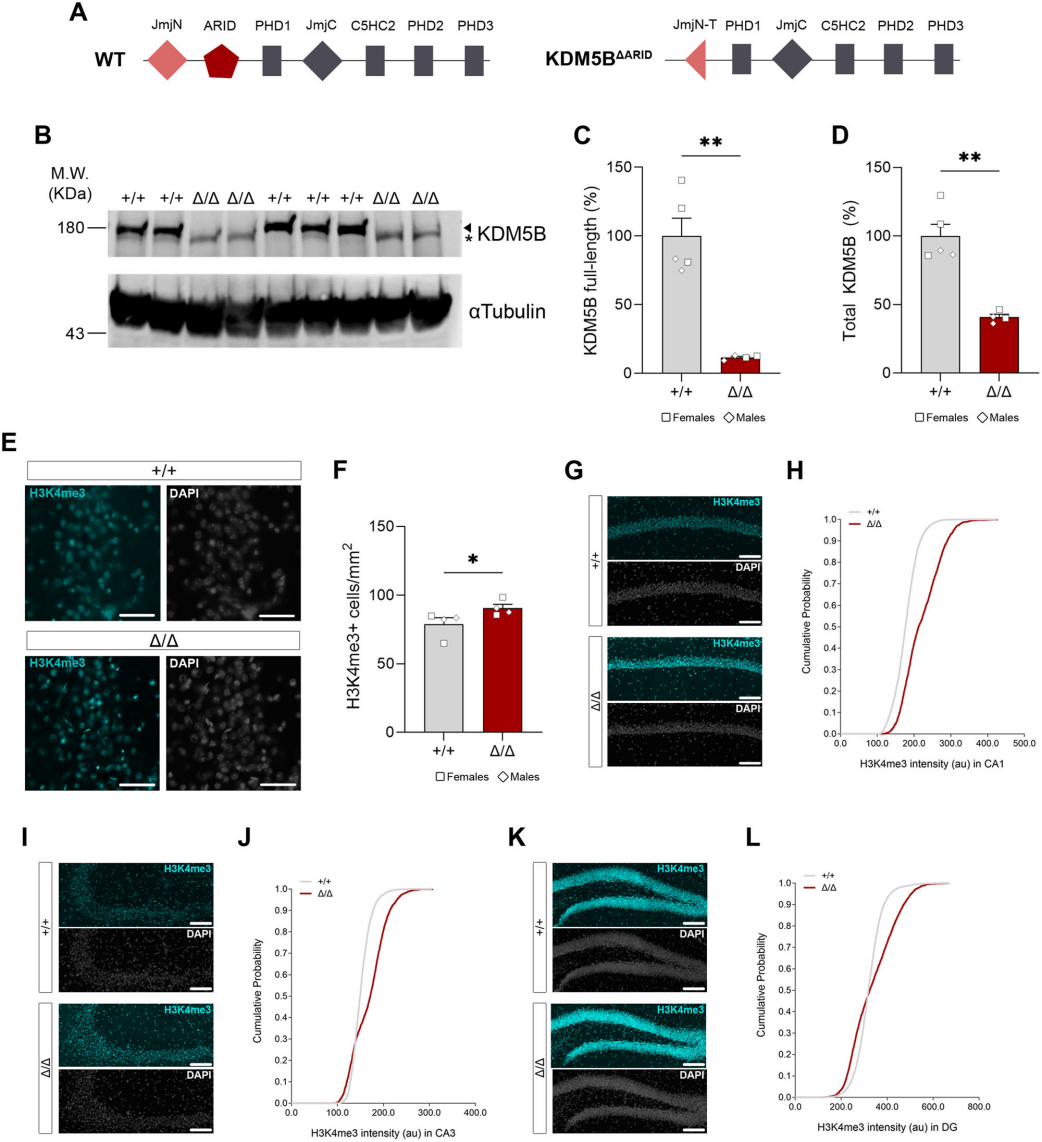
*Kdm5b^Δ/Δ^* mice express reduced total KDM5B levels and show increased number of H3K4me3-positive cells in the hippocampus. ***A***, Schematics of WT and KDM5B^ΔARID^ protein domains. The mutant allele results in a truncated carboxyl end of the JmJN domain (JmjN-T) together with a deletion of the entire ARID domain. ***B***, Representative Western blot images from WT (+/+) and homozygous (Δ/Δ) mutant mice postnatal Day 5 hippocampal samples showing the lack of full-length KDM5B protein in cortical samples from Δ/Δ mice. α-Tubulin was used as a loading control. Full-length KDM5B (arrowhead) and truncated ΔARID (asterisk) protein bands are indicated. See Extended Data Figure 1-1 for uncropped blot. MW markers in kDa are shown on the left. Female (squares) and male (diamonds) samples are included, although no sex differences were observed. ***C,D***, Quantification of full-length and total (full-length and truncated) KDM5B protein from ***B***. ***E,F***, Quantification of the density of H3K4me3+ (cyan) cells in the hippocampus of 8-week-old WT and mutant mice. Nuclei were counterstained with DAPI (gray). Scale bar, 50 µm. Female (squares) and male (diamonds) samples are included, although no sex effect was observed. ***H,J,L***, Cumulative frequencies of H3K4me3-positive cells as a function of their staining intensity. Cumulative probability was calculated including all the detected cells (CA1, 406–903 cells; CA3, 236–925 cells; DG, 925–2,491 cells; 4 animals/genotype). ***G,I,K***, Representative H3K4me3 (cyan) immunostaining of hippocampal sections in 8-week-old control and mutant mice. Sections were counterstained with DAPI (gray). Scale bar, 200 µm. Data in ***C,D,F*** is shown as mean ± SEM and is analyzed with Student's *t* test. *p < 0.05, **p < 0.01.

Brain cortices were dissected from adult mice, and the whole-cell protein was prepared by lysing in 8 M urea, 1% CHAPS, and 50 mM Tris, pH 7.9, lysis buffer containing protease inhibitors. Samples were rotated for 30 min at 4°C and then centrifuged for 50 min to remove DNA. The supernatant was stored at −80°C. All reagents and machinery were obtained from Bio-Rad unless stated otherwise. Samples were prepared with the Laemmli buffer containing 10% β-mercaptoethanol and resolved with 7.5% Mini-PROTEAN precast polyacrylamide gels and Tris/glycine/SDS buffer. A 60 µg sample of protein was used to detect the KDM5B protein in brain lysates. Proteins were transferred to a nitrocellulose membrane with the Trans-Blot turbo system [high molecular weight (MW) transfer program]. Membranes were blocked with 5% BSA (Sigma) diluted in a Tris-buffered saline containing 0.1% Tween 20 (TBS-T). Primary antibodies were diluted in TBS-T containing 5% BSA. Primary antibodies were incubated overnight at 4°C in 5% BSA in TBS-T. Membranes were incubated with secondary antibodies diluted in TBS-T containing 5% BSA for 1 h at room temperature. Proteins were detected with Clarity ECL reagent, and membranes were imaged using the ChemiDoc system. Densitometric analyses were performed with ImageJ software (NIH). The following antibodies were used: anti-KDM5B (Abcam, ab181089, 1:1,000), α-tubulin (Upstate, 05-829, 1:5000), and goat anti-rabbit and anti-mouse HRP secondary antibodies (Thermo Fisher Scientific, #31460, and Proteintech, #SA00001-1, respectively, 1:5,000). Uncropped full scans of the blots depicted in Figure 1*B* are shown in Extended Data Figure 1-1.

10.1523/JNEUROSCI.1544-23.2024.f1-1 Figure 1-1 Uncropped western blot full scans for the corresponding cropped blots on Figure 1B. Download Figure 1-1, TIF file.

### Immunofluorescence

Animals were perfused with either 1× PBS or 1× PBS followed by 4% paraformaldehyde (PFA). Brains were removed and fixed overnight in 4% PFA. Samples were cryopreserved with a sucrose gradient (5, 15, and 30%, 1 d each). Free-floating coronal brain sections (20–30 µm) were obtained with a cryostat. Sections were first washed twice in PBS-T (1× PBS 0.3% Triton X-100) and blocked for 1 h in a blocking buffer (BB: PBS-T 3% NGS 3% BSA). Following blocking, primary antibodies were incubated overnight in BB at 4°C. The following day, slices were washed twice with PBS-T, and secondary antibodies were incubated in BB for 2 h. Samples were washed three times with PBS-T before nuclei staining with Hoechst3332 (1:5,000). Two final washes with 1× PBS were performed before mounting onto glass slides with anti-fade mounting media (Abcam, ab104135). The following antibodies were used (1:200 dilution): Npas4 (Activity Signaling, NP41-2), GFP (Abcam, ab13970), H3K4me3 (Cell Signaling Technologies, 9751S), goat anti-rabbit 488 (Thermo Fisher Scientific, A11031), and goat anti-chicken 488 (Thermo Fisher Scientific, A11039). Images were acquired with a Zeiss Axio Observer 7 microscope (20× magnification) and Zen Blue software. Image acquisition and analysis were performed blindly. The number of Npas4-positive nuclei in the hippocampal layers CA3 and DG was quantified manually with the Cell Counter plug-in on ImageJ-FIFI (NIH). The number/density and mean staining intensity/cell of H3K4me3 were analyzed in cornu ammonis 1/2/3 (CA1/2/3) and the dentate gyrus (DG) with QuPath (Bankhead et al., 2017). The following settings were used to detect the positive cells: requested pixel size, 0.5; background radius, 8; median filter radius, 0; sigma, 1.5; cell expansion, 2 (all in µm); minimum area, 10; maximum area, 400 (in µm^2^); and threshold, 10.

### Golgi staining and spine analysis

Fresh brain hemispheres were processed with the FD Rapid GolgiStain™ kit (FD NeuroTechnologies) following the manufacturer's instructions. Briefly, 100 µm sections were obtained with a vibratome and mounted on gelatine-coated superfrost slides. Following the staining procedure, bright-field images of impregnated dendrites from dorsal hippocampal neurons were captured with a Zeiss Axio Observer 7 microscope (63× magnification) and Zen Blue software. Stacks were taken every 0.2 µm and analyzed manually with Fiji. Spine density was calculated in 10 proximal dendrites per area and animal, starting 5 µm apart from the ramification. Spine density values are shown as average for each area and animal.

### Behavior

**Figure 2.**
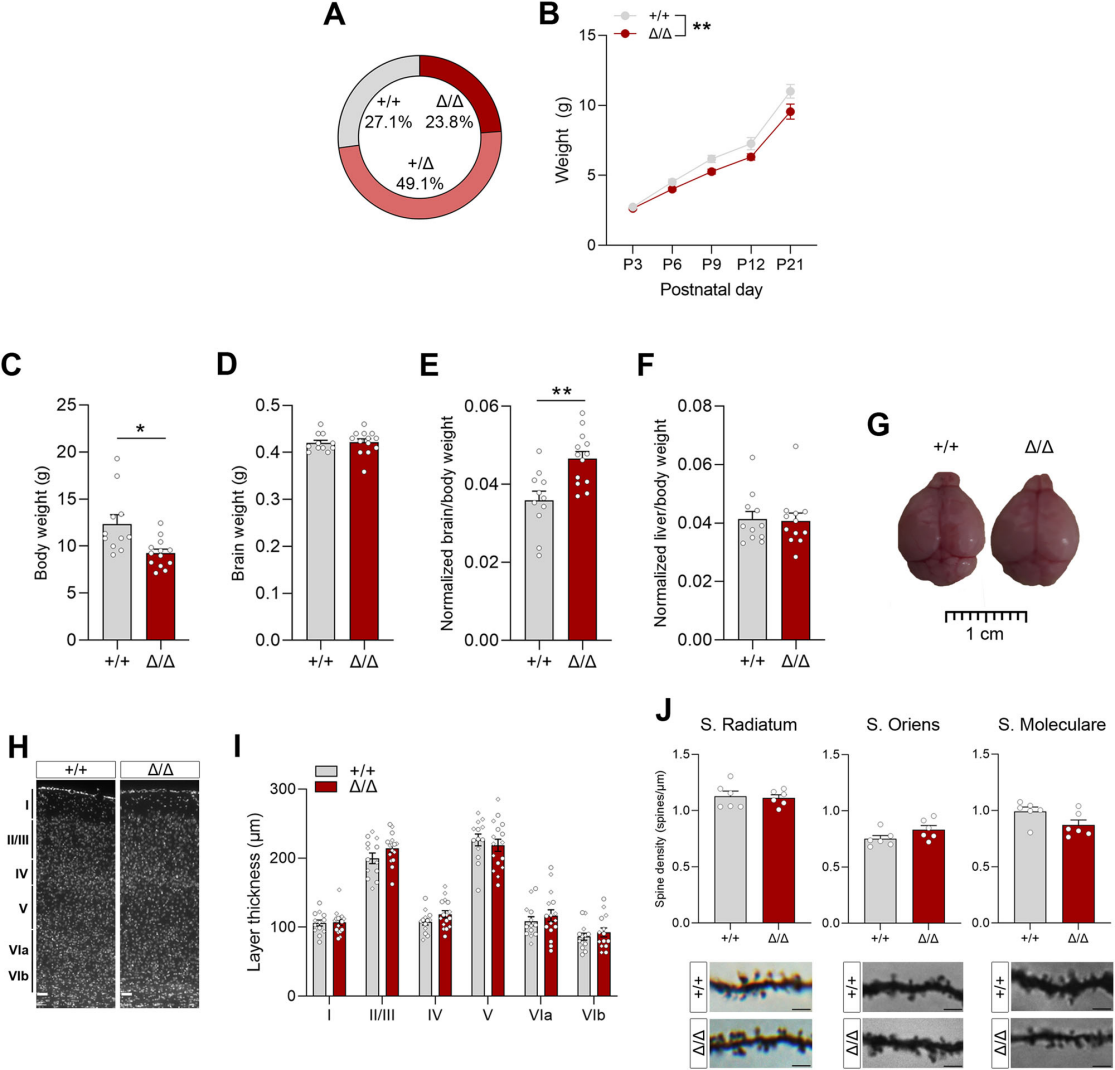
*Kdm5b^Δ/Δ^* homozygous mice are viable but exhibit growth retardation and increased brain:body weight. ***A***, Homozygous mutants are viable and present at expected Mendelian ratios at P21; *n* = 128; chi^2^, *p* = 0.9207. Similar Mendelian rates are observed when analyzing males and females separately (males: 25.38% +/+, 51.52% Δ/Δ, 23.11% Δ/Δ; females: 27.96% +/+, 47.67% Δ/Δ, 24.37% Δ/Δ). ***B***, Preweaning body weight (g) measurements show decreased body weight in mutant animals. ***C–F***, Body and brain weight, and brain:body and liver:body weight ratios are shown for +/+ (*n* = 11) and Δ/Δ (*n* = 13) P21 mice. ***G***, Representative images of brains from WT and homozygous mutant mice indicating similar sizes. ***H***, General cortical brain architecture is not affected in homozygous animals. Representative images of cortical brain sections, where nuclei visualized with Hoechst3332 are shown. Scale bar, 50 µm; *n* = 13 +/+ and *n* = 14 Δ/Δ animals. ***I***, Layer thickness was measured in the somatosensory cortex. ***J***, Golgi-cox–stained proximal basal and apical dendrites of CA1 pyramidal neurons and apical dendrites of DG granule cells in the dorsal hippocampus were analyzed. Graphs depict the average spine density per animal (10–20 dendrites/hippocampal region). Images show representative Golgi-Cox–stained dendrites. Scale bar, 2.5 µm. *N* = 6 mice/genotype. Data is shown as mean ± SEM, including female and male mice. Data was analyzed with two-way ANOVA (***B,I***) or Student's *t* test or Mann–Whitney test when appropriate (***C–F,J***). *p < 0.05, **p < 0.01.

**Figure 3.**
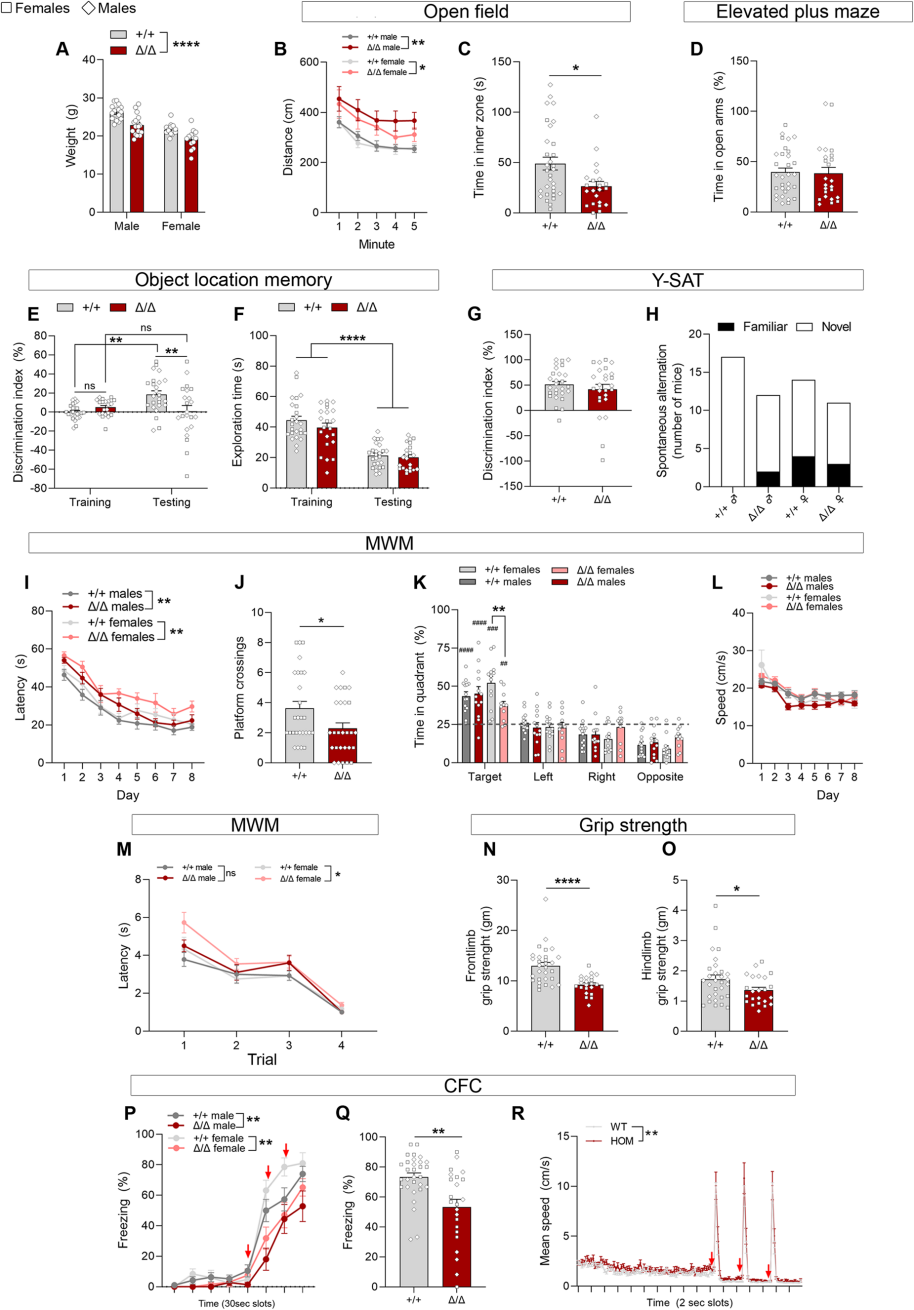
*Kdm5b^Δ/Δ^* homozygous mice exhibit hyperlocomotion and learning deficits. ***A***, Body weight differences in 2-month-old mice before the start of the behavioral tests. Two-way ANOVA genotype effect, ****p < 0.0001; sex effect, ***p < 0.001. ***B***, Distance moved (cm) in the outer zone of the open-field arena. Two-way ANOVA genotype effect: ***p = 0.0005. Three-way ANOVA sex effect: *p* = 0.33. ***C***, The amount of time (s) in the inner zone of the open field is shown. Two-way ANOVA genotype effect, **p = 0.0087; sex effect, *p = 0.0107. ***D***, Time (%) spent in the open arms in the EPM, indicative of reduced anxiety. Two-way ANOVA genotype effect, *p* = 0.8675; sex effect, *p* = 0.4664. ***E***, DI during the training and test (24 h) phases in the OLM test to analyze long-term spatial memory. Note significant learning in WT mice compared with training, but no significant learning in *Kdm5b^Δ/Δ^* mutants, and the reduced DI on testing session between the two genotypes. Two-way ANOVA interaction effect: **p = 0.0057. Two-way ANOVA sex effect: *p* = 0.6317 (training) and *p* = 0.9086 (testing). ***F***, There are no genotype differences in total exploration time (s) during training nor testing session in the OLM test. Two-way ANOVA genotype effect, *p* = 0.2105 (training) and *p* = 0.5919 (testing), and sex effect, *p* = 7,506 (training) and *p* = 0.2431 (testing). ***G***, DI during the testing session of the Y-SAT, performed 1 h after training to assess short-term spatial memory. Two-way ANOVA sex effect: *p* = 0.0768. ***H***, Spontaneous alternation rate in the Y-SAT shows no differences between genotypes. ***I***, Latency (s) to reach the hidden platform during the training phase of the MWM. Data represents the mean of the four trials/day. Two-way ANOVA genotype effect: ***p = 0.0001. Three-way ANOVA sex effect: *p* = 0.0019. ***J,K***, Graphs show the number of platform crossings (***J***) and time spent (%) in the different quadrants (***K***) during the probe trial performed on Day 8. Chance, 25%, is depicted with a dashed line. Two-way ANOVA sex effect in ***J***: *p* = 0.3413. ***L***, Swimming speed (cm/s) was similar between genotypes during the training phase of the MWM. Three-way ANOVA sex effect, *p* = 0.0757; genotype effect, *p* = 0.1008. ***M***, Latency (s) to find the platform during the visible phase of the MWM. Three-way ANOVA genotype effect, *p = 0.0220; sex effect, *p* = 0.1982. ***N,O***, Front- and hindlimb grip strength (gm) was tested three times and the average is shown. Two-way ANOVA sex effect: *p* = 0.1118 (***N***) and *p* = 0.1924 (***O***). ***P***, Freezing behavior was assessed during CFC training, when three shocks were administered. Three-way ANOVA sex effect, *p* = 0.07; genotype effect, ****p < 0.0001. ***Q***, Freezing percentages in the context testing session, 24 h later. Two-way ANOVA sex effect: *p* = 0.1489. ***R***, Mean speed was higher in mutant mice, in line with their reduced freezing behavior during training (two-way ANOVA genotype effect: **p = 0.0053). However, no differences were observed between genotypes during the 2-s-long foot shocks [Tukey's post hoc test: *p* = 0.9943 (shock 1); *p* > 0.9999 (shock 2); *p* = 0.9998 (shock 3)], thus discarding differences in sensitivity and responses to shocks as a contributing factor. Data was analyzed with two-way ANOVA (***A,E,F***) or repeated measures two-way ANOVA (***B,I,L,M,P,R***) followed by Tukey's post hoc test. Data in ***C,J,K,N,O,Q*** was analyzed with Student's *t* test, or Mann–Whitney test when appropriate. *p < 0.05, **p < 0.01, ***p < 0.001, ****p < 0.0001. A one-sample *t* test was used (***K***) to analyze whether time spent in the target quadrant was above chance. ^##^*p* < 0.01, ^###^*p* < 0.001, ^####^*p* < 0.0001. For all experiments, *n* = 17 +/+ male, *n* = 14 +/+ female, *n* = 17 Δ/Δ male, and *n* = 13 Δ/Δ female mice. Female (squares) and male (diamonds) samples are included.

**Figure 4.**
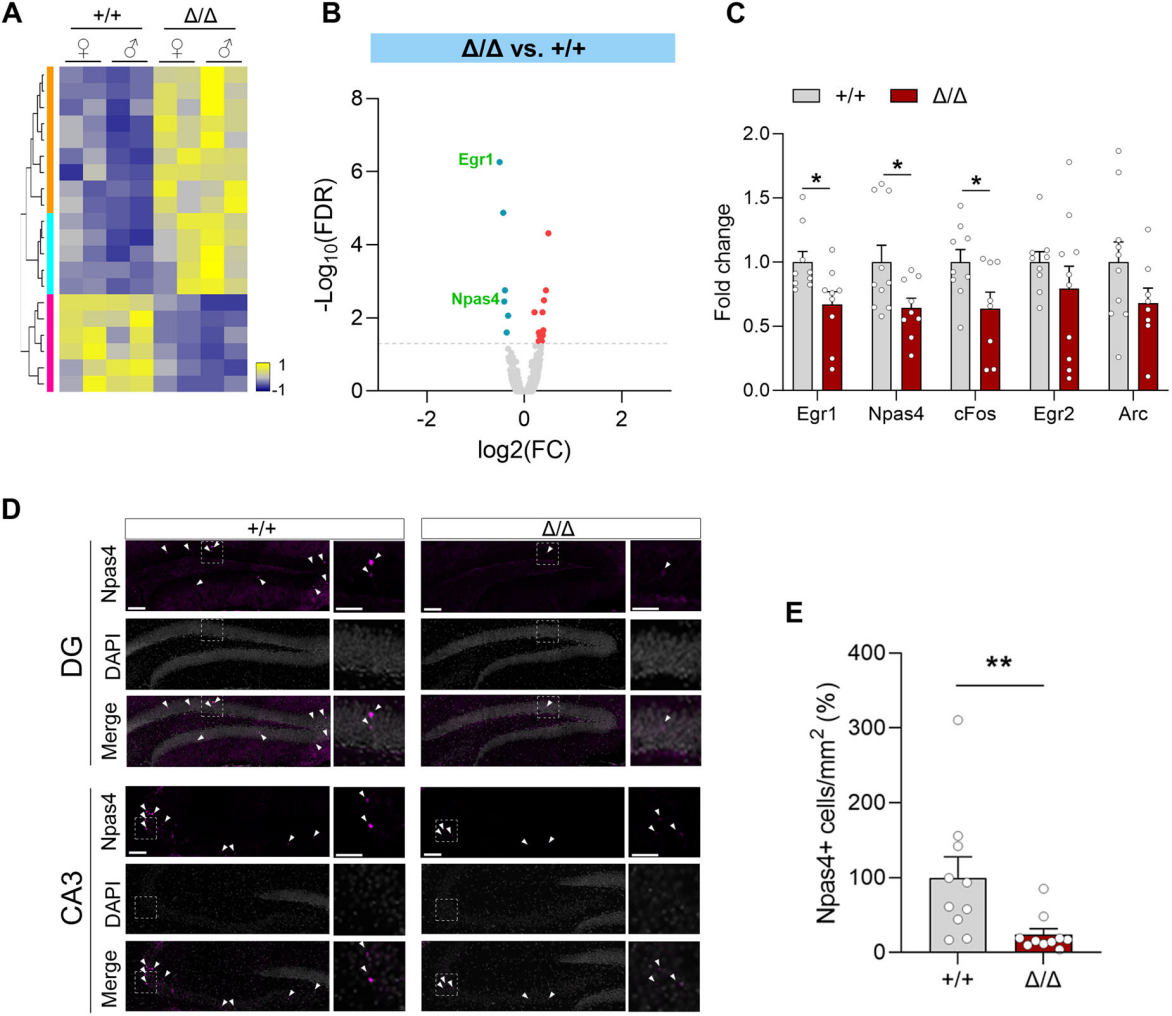
Altered gene expression in *Kdm5b^Δ/Δ^* mice. Hippocampi were dissected from naive animals and RNAseq analyses were performed (*n* = 4 animals/genotype). ***A***, Heatmaps show DEGs in WT control (+/+) and mutant (Δ/Δ) mice. Yellow, upregulated, and blue, downregulated in mutants. See Extended Data Table 4-1 for detailed RNA-sequencing data information. ***B***, Volcano plot displaying baseline gene expression changes between WT and *Kdm5b^Δ/Δ^* homozygous mutant mice. Red and blue dots depict differentially up- and downregulated genes, respectively. Differentially expressed immediate early genes are labeled. ***C***, Immediate early gene expression in the dorsal hippocampus from WT and *Kdm5b^Δ/Δ^* mice was analyzed by qPCR; *n* = 9 animals/genotype. Two-way ANOVA genotype effect, ***p = 0.0001. ***D***, Immunostaining of sections from DG and CA3 with an Npas4-specific antibody (magenta), counterstained with DAPI (gray), is shown. White arrowheads indicate positive nuclei. ***E***, The number of Npas4-positive cells (arrowheads) was quantified in mm^2^ areas of the CA3 and DG. Scale bar, 200 µm, 100 µm for higher magnification images; *n* = 10 mice/genotype. Data is shown as mean ± SEM, including both female and male mice. Data was analyzed with Student's *t* test (***C,E***): *p < 0.05, **p < 0.01.

**Figure 5.**
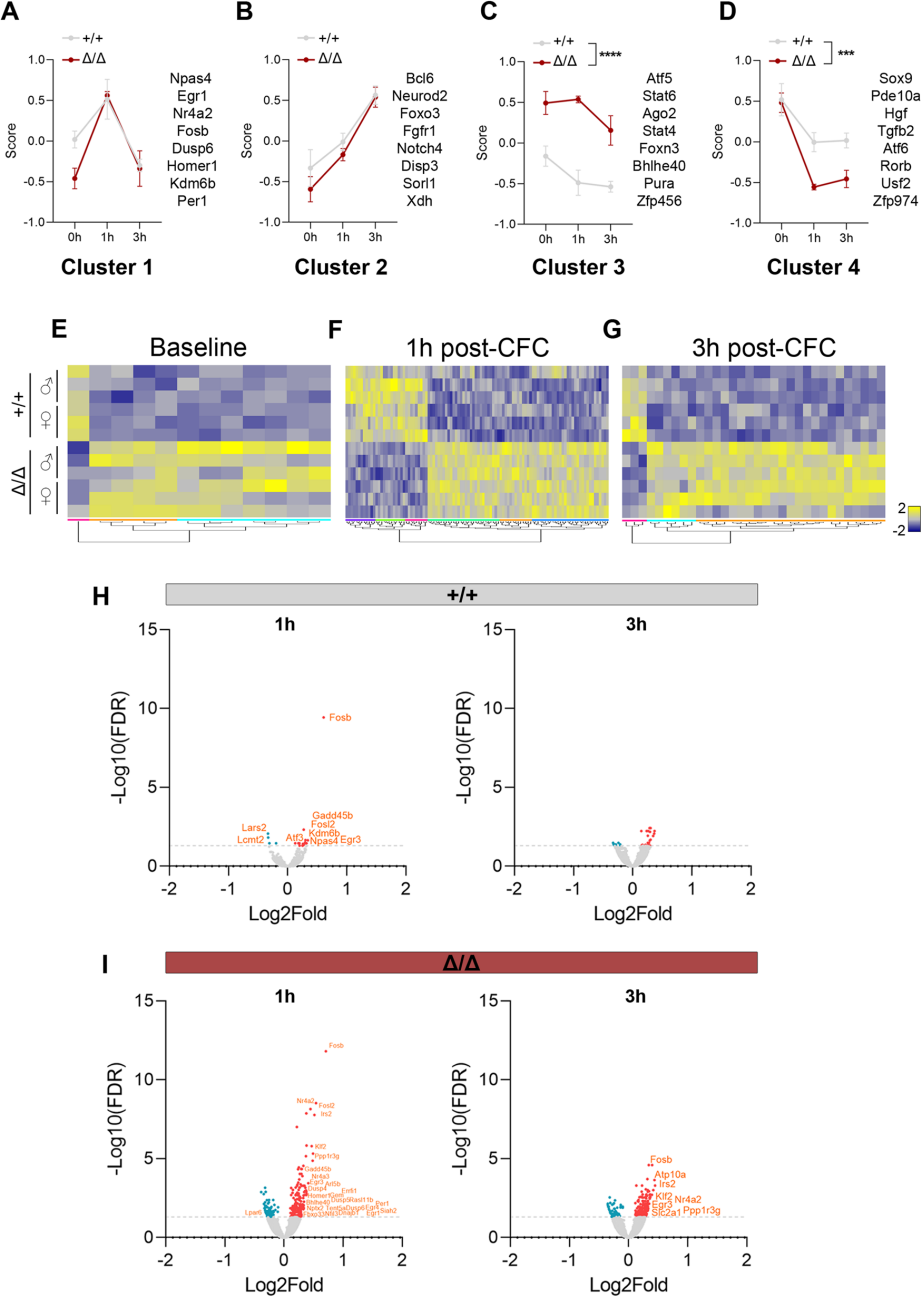
Learning-associated gene expression changes in *Kdm5b^Δ/Δ^* mice. WT (+/+) and *Kdm5b^Δ/Δ^* (Δ/Δ) mice were trained in the fear-conditioning chamber and culled 1 or 3 h later. Home cage, test-naive mice were used as controls. The dorsal hippocampus was dissected and RNAseq analyses were performed. *n* = 3 males and 3 females per genotype and timepoint. ***A–D***, DEGs were clustered by their expression trajectories with *k*-means clustering (*k* = 4). Selected genes within each cluster are shown on the right. ***E–G***, Heatmaps show DEGs in control and mutant mice at baseline levels (***E***) or 1 h (***F***) and 3 h (***G***) after fear conditioning. Yellow, upregulated, and blue, downregulated. ***H,I***, Volcano plots display gene expression changes after conditioning. Red and blue dots depict differentially up- and downregulated genes, respectively, at 1 or 3 h compared with control animals for each genotype. Differentially expressed activity-regulated genes are labeled in orange. Data is shown as mean ± SEM, including female and male mice. Data was analyzed with repeated measures two-way ANOVA (***A–D***): ***p < 0.001, ****p < 0.0001. See Extended Data Figure 5-1, Table 5-1, and Table 5-2.

**Figure 6.**
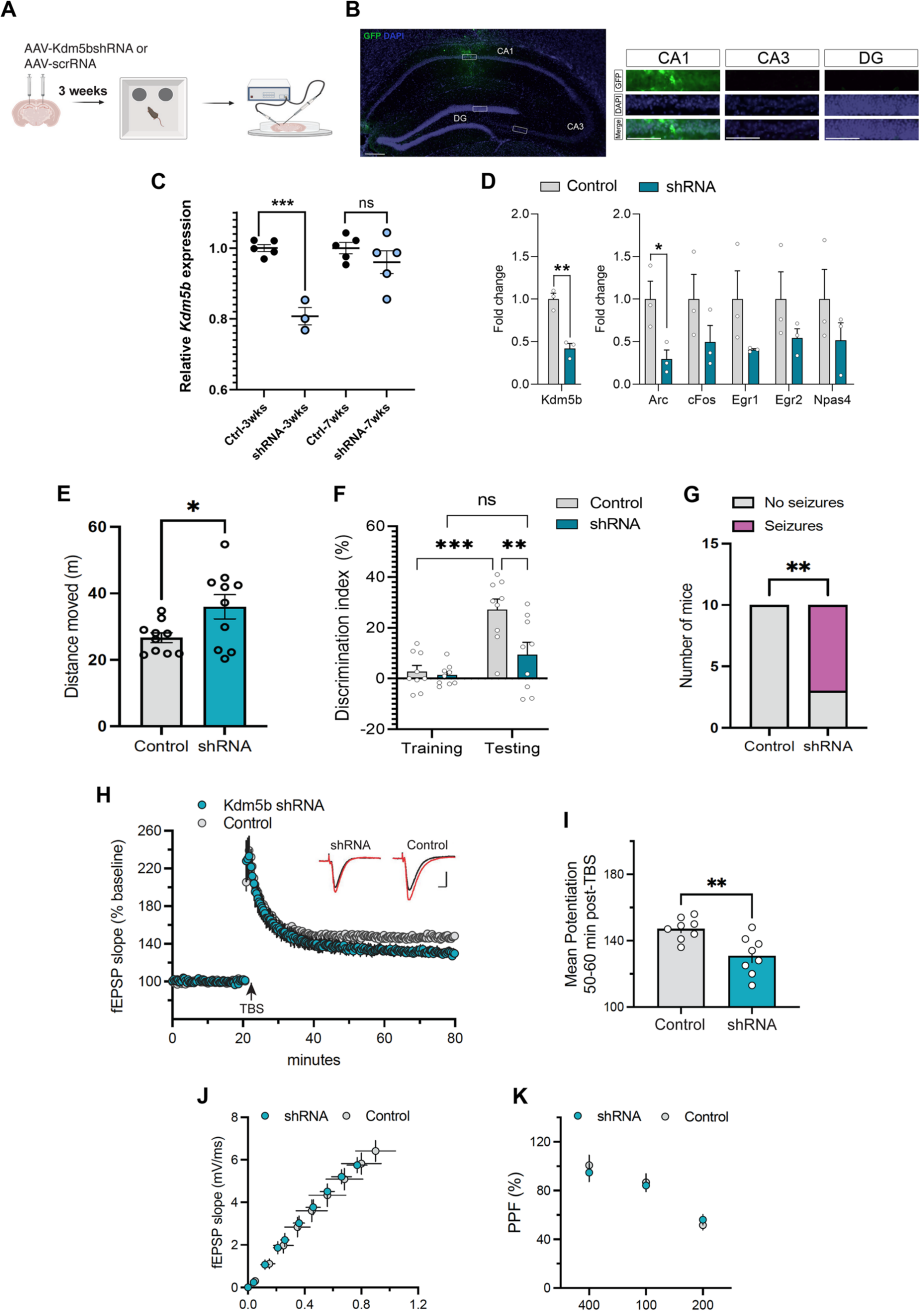
*Kdm5b* knockdown in the dorsal hippocampus (CA1) of adult mice abrogates hippocampus-dependent memory consolidation and diminishes LTP. ***A***, Diagram of experimental workflow. Approximately 3 weeks after stereotactic viral delivery into CA1, mice were habituated and tested in the OLM task. Upon completion of behavioral tests, synaptic plasticity was assessed in acute brain slices from these mice. ***B***, Representative immunostaining of the dorsal hippocampus transduced with GFP-expressing AAV1, 11 d after surgery. Scale bar, 200 µm. Panels display representative CA1, CA3, and DG inset images with GFP (green) and DAPI (blue) labeling. Scale bar, 50 µm. ***C***, qRT-PCR analysis of *Kdm5b* expression, relative to *Hprt*, from the total RNA extracted from the dorsal hippocampus at indicated times (3 and 7 weeks after viral delivery). *N* = 3–5 mice/group. ***D***, qRT-PCR analysis of *Kdm5b* and immediate early gene expression, relative to *Hprt*, from the total RNA extracted from the dorsal hippocampus, 11 d post-transduction. Two-way ANOVA shRNA effect for IEGs: **p = 0.0016. *N* = 3 mice/group. ***E***, Distance moved (cm) in the test arena (open field) on the first day of habituation as a measure of general activity (*n* = 10 control and *n* = 10 shRNA females). ***F***, DIs in the OLM task for control and shRNA mice during training and 24 h long-term memory tests are shown. Note significant learning in control mice compared with training, but no significant learning in shRNA mice compared with controls (*n* = 9 control and *n* = 9 shRNA female mice). Two-way ANOVA interaction effect: *p = 0.0257. ***G***, Number of mice of each group (*n* = 10 each) showing spontaneous seizures during handling (pink) compared with no seizures (gray). Fisher's exact test: **p = 0.0031. ***H***, Short- and long-term plasticity changes measured from hippocampal area CA1b apical dendrites in acute hippocampal slices (*n* = 8 slices from each group; *n* = 4 mice/group). Following a 20 min stable baseline recording, TBS (arrow) was delivered to induce LTP, and recordings were followed for an additional 1 h. The fEPSP slope measured from *Kdm5b* shRNA slices was noticeably lower relative to controls by the end of the recording period. Inset: representative traces collected during baseline (black line) and 60 min post-TBS (red line). Scale, 1 mV/5 ms. ***I***, The mean potentiation 50–60 min post-TBS was significantly reduced in slices from *Kdm5b* shRNA mice relative to controls (**p = 0.0056; *n* = 8 each). ***J,K***, Short-term plasticity measures in slices from *Kdm5b* shRNA mice including the (***J***) i/o curve and (***K***) paired-pulse facilitation did not reveal any significant differences from shScramble controls. Data are shown as mean ± SEM, analyzed with Student's *t* test (***C,D,E,I***), two-way ANOVA (***F***), and Fisher's exact test (***G***). *p < 0.05, **p < 0.01, ***p < 0.001. Control, shScramble; shRNA, shKdm5b.

Different cohorts of control and *Kdm5b*-mutant mice were used for the experiments in this study: (1) battery of behavioral tests (Fig. 3), (2) confirmation of the anxiety-related phenotype, (3) RNAseq with naive animals (Fig. 4*A,B*), (4) confirmation of the RNAseq results by immunofluorescence and RT-qPCR (Fig. 4*C–E*), and (5) RNAseq to study gene expression changes upon learning (Fig. 5). For the *Kdm5b* knockdown experiment (Fig. 6), a cohort of mice was used for the behavioral and the electrophysiology analyses, and extra cohorts were used to confirm the *Kdm5b* knockdown 1, 3, and 7 weeks after the surgery. A final cohort was dedicated to study the effect of the knockdown on early gene expression 11 d after the surgery.

Behavioral assessments of F2 mice (Cohort 1) started at 7–8 weeks of age, consisting of the following: handling, open field test, object location memory (OLM) test, elevated plus maze (EPM), spontaneous alternation in a Y-maze test, Morris water maze (MWM), fear-conditioning test, and measurement of grip strength. Mice were left to rest for at least 2 d in between tests. Mice in Cohorts 3, 4, and 5 were 7–8 weeks old when samples were collected.

Behavioral experiments were conducted between 8:00 and 18:30 under standard room lighting conditions unless stated otherwise. Cages were changed every 2 weeks but never <48 h before the day of testing. Behaviors were tracked using EthoVision (Noldus Information Technologies). After each trial of a specific test, boli and urine were removed, and the test area was cleaned with 1% Anistel® (high-level surface disinfectant, Trisel Solution, for Cohort 2) or 10% ethanol (Cohort 1) to remove any odors. The experiments were blinded and randomized by blocks of mice. Littermates were used as controls with multiple litters examined per experiment (15 for Cohort 1 and 12 for Cohort 2). Mice were habituated to the behavioral room conditions for at least 30 min before the start of each testing session. Before the start of the experiments, mice were individually handled for 5 consecutive days, 2 min each day.

Behavioral assessments of shRNA mice were started 2–3 weeks after stereotactic surgeries. Mice were first tested in the EPM to determine effects on activity and anxiety, handled for 5 d as described and habituated to the test arena for 6 d before training with two identical objects for 10 min, followed by a 5 min testing session 24 h later with one of the objects moved to a new location (Kwapis et al., 2018). Two weeks later, the tissue was collected for acute slice preparations and LTP.

#### Open field

The circular open-field arena was made of clear acrylic with a gray base, with internal dimensions of 40 cm in diameter and 40 cm high as described (Whittaker et al., 2017). The light intensity inside the arena was 10 lux. Two virtual areas were drawn on EthoVision: a center zone, 20 cm in diameter, and a ring, 5 cm thick around the perimeter of the arena, which was defined as the outer zone. Test mice were placed in the open field facing an outer wall to begin the test. Its locomotor activity was tracked for 5 min. The total distance moved (cm) in the outer zone was used as a measure of the level of locomotive activity and time (s) spent in the center zone used as a measure of anxiety.

#### OLM test

The test was performed as described in Vogel-Ciernia et al. (2013). The arena (40 × 40 × 40 cm) was made of white acrylic and had a black stripe in one of the walls as an internal cue. The light intensity was 40 lux in the center of the arena. Briefly, mice were habituated to the arena for 6 consecutive days, 5 min/day. During the training session, mice were placed for 10 min in the arena with the two identical objects (100 ml glass beakers filled with cement to prevent them from being moved by the mice) next to the wall with the visual cue. Twenty-four hours later, during the testing session, one of the two objects was randomly moved to the opposite side of the arena, in a center position, and mice were left to explore the arena for 5 min. Exploration was manually scored, and the following criterion was used: nose of the mouse is within 1 cm of the object, with their head directly facing it. Rearing, standing on the object, and digging near it were not considered as exploratory behaviors. The discrimination index (DI) was calculated as (time exploring new location – time exploring old location) / (total exploration time) * 100. Exclusion criteria included exploration time below 3 s for either training or testing sessions, and DI above +/−20 for the training session.

#### EPM

The EPM was made of black acrylic and consisted of four arms (30 × 5 cm). The two opposing closed arms were enclosed by 15 cm high walls on each side and ends. The two opposing open arms were open, as well as the center platform (5 × 5 cm). The maze was elevated 40 cm aboveground. The light intensity was 100 lux on the open arms and 10 lux on the closed arms. The number of entries onto, time spent on, and latency to enter the closed and open arms was manually scored. An arm entry is defined when all four paws were located inside the arm. Mice were placed in the center platform of the EPM facing a closed arm to start the 5 min test.

#### Spontaneous alternation in a Y-maze

The Y-maze apparatus was made of gray acrylic and consisted of three arms, 120° from each other. All arms were 45 cm long and 5 cm wide and enclosed by a 10 cm wall. Extramaze visual cues were placed at the end of each arm. One arm was considered as the central arm, and the other two arms were randomly closed during the training session. During the training session, mice were placed in the maze in the central arm and allowed to explore it for 10 min, after which they were returned to their home cage. After 1 h, the testing session was conducted. Both arms were open, and mice were placed in the central arm and allowed to freely explore the maze for 5 min. The first choice to turn either to the familiar arm or the new arm (percentage of alternation rate) was monitored, when all four paws were inside that arm. Arm preference was automatically monitored with EthoVision, and the DI was calculated as (time exploring new arm – time exploring old arm) / (total exploration time of the old and new arms) * 100.

#### MWM

Spatial learning and memory were assessed using a MWM. The maze (1 m in diameter) was filled with water and made opaque with a nontoxic white aqueous emulsion. The water temperature was 23°C, and the light intensity was 100 lux. A set of extramaze visual cues were suspended around the pool. Four alternative start positions were nominated to provide the virtual division of the tank into four quadrants. Mice were trained to find a hidden platform (10 cm in diameter) and submerged 2 cm below the water surface, for 8 consecutive days, four trials a day. Once they found the platform, mice were left on it for 15 s before returning them to their home cage. Mice that failed to locate the hidden platform after 60 s were placed on the platform for 15 s. Animals that failed to stay on the platform following 2 d of training were excluded from the study. On the probe trial, the platform was removed, and mice were placed into the pool for 60 s so they could freely explore the tank. To check for deficiencies in vision or locomotion, mice were placed in the pool with a visible platform (1 cm above the surface, with a flag attached to it), 1 d, four trials. The mouse movement was tracked with EthoVision to calculate the mean speed, the total distance traveled in the pool and in each quadrant, and the number of platform crossings during the probe trial.

#### Fear-conditioning test

Animals were placed in a soundproof fear-conditioning apparatus with stainless steel metal grid floor, containing a camera (Med Associates). To provide an olfactory cue, an ethanol-soaked tissue was placed under the grid in both training and testing sessions. The mouse behavior was recorded with VideoFreeze software. For conditioning, mice were placed inside the chamber and left to freely explore it. After 148 s, three electric shocks (0.7 mA, 2 s each, 30 s apart) were administered. The animals were then removed from the testing chamber after 30 additional seconds. 24 h later, mice were placed in the same chamber for 5 min. The freezing behavior was manually scored from recorded videos during training and testing sessions. The freezing behavior was defined as the complete lack of movement during the first 2 s of each 5 s window.

#### Grip strength measurements

To assess the neuromuscular ability of the animals, the fore- and hindlimb grip strength was measured with a Linton Grip Strength Meter (MJS Technology). Mice were pulled across the meter from left to right measuring the forelimb and then the hindlimb strength. The average of three measurements per limb and mouse was taken.

### RNA extraction

The total RNA was extracted from hippocampal samples with 1 ml TRIzol (Thermo Fisher Scientific) and further purified with the Monarch Total RNA Miniprep Kit (New England Biolabs) following the manufacturer's recommendations (including DNAse treatment step).

### qRT-PCR analysis

cDNA was synthesized from 200 to 400 ng RNA with UltraScript 2.0 cDNA Synthesis Kit (PCR Biosystems) according to the manufacturer's instructions. qRT-PCRs were performed on a Bio-Rad CFX384 using qPCRBIO SyGreen Mix Lo-ROX (PCR Biosystems). Relative expression levels were calculated using the 2^−ΔΔCT^method and *Hprt* or *Gapdh* was used as the endogenous control gene. For shRNA experiments, the PrimeTime probe-based gene expression system (IDT) was used to quantify *Kdm5b* expression, relative to *Hprt*. Primer sequences are as follows: *Arc* (Fw, 5′-CTCAACTTCCGGGGATGCAG-3′; Rv, 5′-CTGGTATGAATCACTGGGGGC-3′), *cFos* (Fw, 5′-AGAGCGGGAATGGTGAAGAC-3′; Rv, 5′-AGTTGATCTGTCTCCGCTTGG-3′), *Egr1* (Fw, 5′-TGAGCACCTGACCACAGAGTC-3′; Rv, 5′-TAACTCGTCTCCACCATCGC-3′), *Egr2* (Fw, 5′-GTGCTGCCTGACAGCCTCTA-3′; Rv, 5′-TTGATCATGCCATCTCCCGCC-3′), *Gapdh* (Fw, 5′-AGGTCGGTGTAACGGATTTG-3′; Rv, 5′-TGTAGACCATGTAGTTGAGGTCA-3′), *Hprt* (Fw, 5′-GTCCCAGCGTCGTGATTAGC-3′; Rv, 5′-TGGCCTCCCATCTCCTTCAT-3′), *Kdm5b* (Fw, 5′-AAGCCAAGCTCTGTTCAGCAA-3′; Rv, 5′-GAAGGCAATCGTTCTTCTCACT-3′), *Npas4* (Fw, 5′-CTGCATCTACACTCGCAAGG-3′; Rv, 5′-GCCACAATGTCTTCAAGCTCT-3′), *Kdm5b* (for Fig. 6, Fw, 5′-CAAGAGCCCACTGAGAAGAAA-3′; Rv, 5′-TCCACATAAGAGGCACACATAC-3′), and *Hprt* (for Fig. 6, Fw, 5′-TGCTCGAGATGTCATGAAGG-3′; Rv, 5′-CTTTTATGTCCCCCGTTGAC-3′).

### RNAseq

Total RNA (*n* = 4/genotype, balanced for sex) were sent to Novogene for library preparation and sequencing. After mRNA enrichment, mRNA quality was analyzed using Agilent Total RNA 6000 Pico on a Bioanalyser (Agilent, 2100). Pair-end sequencing (150 bp read length) was performed on the Illumina NovaSeq 6000 platform. Further data analyses were performed using the Galaxy Europe server (https://usegalaxy.eu; https://academic.oup.com/nar/article/50/W1/W345/6572001). The quality of the raw data was checked using FastQC (v0.11.9). Reads were aligned to the mouse genome (mm10) using RNA STAR (v2.7.8a), and aligned reads were counted using featureCounts (v2.0.1). Differential expression analyses were performed using DESeq2 (v2.11.40.7). Multiple comparisons were controlled for using an FDR < 0.05. Exact *p*-values and adjusted *p*-values for all differentially expressed genes (DEGs) are listed in Extended Data Table 4-1 and Table 5-1. Genes with adjusted *p*-value <0.05 were considered as DEGs. Heatmaps were generated with the R package pheatmap. Volcano plots were generated in GraphPad Prism 9.4.1. DEGs in at least one comparison (any timepoint between genotypes, or 1 h/0 h, 3 h/0 h, and 3 h/1 h within the same genotype) were clusterized with *k*-means clustering and selecting the optimal cluster number for the dataset (*k* = 4). Activity-induced genes were obtained from P. B. Chen et al. (2017), with only differentially upregulated transcripts detected by both RNAseq and TRAPseq included. Gene ontology analyses were conducted using g:Profiler (https://biit.cs.ut.ee/gprofiler/gost), where GO molecular function and GO biological process of size 0–1,500 were checked. The applied threshold was “Benjamini–Hochberg FDR < 0.05.”

10.1523/JNEUROSCI.1544-23.2024.t4-1 Table 4-1 Extended RNA-sequencing data of naïve mice as in Figure 4. Hippocampi were dissected from wild-type and *Kdm5b^Δ/Δ^* naïve animals and RNAseq analyses were performed (n = 4 animals/genotype). Download Table 4-1, XLSX file.

### Electrophysiology

Female mice (*n* = 4/treatment, 8 total) were killed for electrophysiology and hippocampal slices prepared as described previously (Vogel-Ciernia et al., 2013). Mice were anesthetized and decapitated, and the brains were rapidly removed into ice-cold, oxygenated dissection medium containing (in mM): 124 NaCl, 3 KCl, 1.25 KH_2_PO_4_, 5 MgSO_4_, 0 CaCl_2_, 26 NaHCO_3_, and 10 glucose. Hippocampal slices (320 µm, coronal) were cut from a vibratome (Leica, Model:VT1000S) before transferring to an interface recording containing prewarmed (31 ± 1°C) artificial cerebrospinal fluid composed of (in mM): 124 NaCl, 3 KCl, 1.25 KH_2_PO_4_, 1.5 MgSO_4_, 2.5 CaCl_2_, 26 NaHCO_3_, and 10 glucose. Slices were perfused continuously at a rate of 1.75–2 ml/min, while the surface of the slices was exposed to warm, humidified 95% O_2_/5% CO_2_. Recordings began following at least 2 h of incubation.

Field excitatory postsynaptic potentials (fEPSPs) were recorded from CA1b stratum radiatum apical dendrites using a glass pipette filled with 2 M NaCl (2–3 MΩ) in response to orthodromic stimulation (twisted nichrome wire, 65 µm in diameter) of Schaffer collateral–commissural projections in CA1c stratum radiatum. Pulses were administered 0.05 Hz using a current that elicited a 50% maximal spike-free response. After maintaining a stable baseline (20 min), LTP was induced by delivering five “theta” bursts, with each burst consisting of four pulses at 100 Hz separated by 200 ms (i.e., theta burst stimulation or TBS). The stimulation intensity was not increased during TBS. Data were collected and digitized by NAC 3.0 (Neurodata Acquisition System, Theta Burst) and stored on a disk.

Data in the text are presented as means ± SD, while in the figures as mean ± SEM. The fEPSP slope was measured at 10–90% fall of the slope, and data in figures on LTP were normalized to the last 20 min of baseline.

### Statistics

Data are reported as mean ± SEM. Graphs show individual data points. Normal distribution was tested with d’Agostino and Pearson’s omnibus, Shapiro–Wilk, and Kolmogorov–Smirnov normality tests. If the test was passed, statistical analysis was performed using parametric statistical analyses. Before pairs of comparisons, we performed the *F* test to compare variances. In experiments with normal distribution, statistical analyses were performed using the unpaired two-sided Student's *t* test. *T*-test with Welch's correction was applied when variances were unequal. Two- and three-way ANOVA with the appropriate post hoc tests were also performed as indicated in the figure legends. Significant *p*-values (*p* < 0.05) are reported in the results section, and/or figure legends provide details of relevant statistical parameters, including group sizes. Statistical analyses were performed with GraphPad Prism (version 9.4.1). Experiments in this study were blinded and animals randomized for in vivo studies.

## Results

### KDM5B demethylase-deficient mice exhibit megalencephaly

*Kdm5b^ΔARID/ΔARID^* (*Kdm5b^Δ/Δ^*) mice were generated to circumvent the embryonic and early postnatal lethality of other loss-of-function models. The deletion of exons 2–4 leads to a partial truncation of the JmjN domain and the deletion of the ARID domain of the KDM5B protein (Fig. 1*A*), which disrupts its H3K4me3 demethylase activity (Jamshidi et al., 2021). We confirmed the loss of full-length KDM5B protein and the appearance of a shorter KDM5B-ΔARID protein in the brain of adult *Kdm5b^Δ/Δ^* mice by Western blot (Fig. 1*B,C*). The mutant KDM5B-ΔARID protein appears to be either unstable or not as well detected by KDM5B-specific antiserum in Western blot, leading to an apparent reduction in total KDM5B protein levels in the homozygous mutants compared with WT mice (Fig. 1*D*). We did not observe differences between male and female animals.

To assess the impact on H3K4me3, hippocampal sections from *Kdm5b^Δ/Δ^* and control mice were immunostained with an antibody to H3K4me3. We observed a clear increase in the number of H3K4me3-positive cells in the hippocampus (Fig. 1*E,F*). This increase was observed in both sexes (two-way ANOVA sex effect: *F*_1,4 _= 0.1824; *p* = 0.6913). Moreover, the intensity of H3K4me3 across the population of cells in the hippocampus shifted toward increased H3K4me3 in the mutants in all hippocampal subregions, CA1 (Fig. 1*G,H*), CA3 (Fig. 1*I,J*), and DG (Fig. 1*K,L*).

In an attempt to further reduce any developmental abnormalities associated with KDM5B deficiency, all animals used in this study were on a C57BL/6Jx129S2/Sv F2 genetic background (Zou et al., 2014; data from the International Mouse Phenotyping Consortium: http://www.mousephenotype.org/). Homozygous *Kdm5b^Δ/Δ^* mice generated from heterozygous intercrosses were present at expected ratios at P21 (Fig. 2*A*). Postnatal *Kdm5b^Δ/Δ^* mice displayed a growth deficit and reduced body weights (Fig. 2*B*). Homozygous mutants exhibited increased brain-to-body weight ratios at postnatal day 21, while the liver-to-body weight ratios did not differ between genotypes, suggesting specific neurodevelopmental alterations (Fig. 2*C–G*). Histological assessments did not reveal any gross abnormalities in the cortical architecture (Fig. 2*H,I*) or spine density in proximal dendrites of CA1 pyramidal neurons or DG granule cells in the hippocampus (Fig. 2*J*).

### Hippocampus-dependent learning and memory deficits in *Kdm5b^Δ/Δ^* mice

To determine whether KDM5B loss of function affects learning and memory, we tested these mice in hippocampus-dependent tasks. Data from males and females were combined, unless mice of different sexes exhibited differences in specific tests, where the data from both sexes are shown. During the handling sessions, we observed that the reduced body weight observed at preweaning stages was still evident at 2 months of age (Fig. 3*A*). *Kdm5b^Δ/Δ^* mice exhibited hyperactivity in the open field (Fig. 3*B*). They also spent less time in the inner area of the arena, suggesting an anxiety-like phenotype (Fig. 3*C*). However, this phenotype was not observed in the EPM in the same cohort of mice (Fig. 3*D*) and was not consistent in a second cohort [for that cohort: distance traveled in the open-field genotype effect, *p* = 0.9966 (males: WT 28.5±7.7, mutant 14.6±3.7; females: WT 12.3±3.7, mutant 26.2±4.5); time (%) spent in the open arms in the EPM genotype effect, *p = 0.0385 (males: WT 10.1±2.9, mutant 13.3±3.6; females: WT 4.2±1.6, mutant 14.9±3.8)], suggesting that the anxiety-like phenotype is variable and potentially secondary to hyperactivity. Next, we performed the OLM test to study long-term spatial memory. A significant deficit in long-term spatial memory was observed in the mutant mice 24 h after training (Fig. 3*E*). Exploration times were equal between genotypes in both training and testing sessions (Fig. 3*F*), indicating a specific deficit in OLM. In contrast, short-term spatial memory and cognitive flexibility were preserved in *Kdm5b^Δ/Δ^* mice, as judged, respectively, by the DI and spontaneous alternation percentage in the spontaneous alternation test in a Y-maze (Y-SAT; Fig. 3*G,H*). Next, we examined long-term spatial learning and reference memory using the MWM. During the training sessions with the hidden platform version of the test, the performance improved significantly in both groups, although escape latency in mutant mice remained significantly higher compared with WT littermates (Fig. 3*I*). During the probe trial on day 8, *Kdm5b^Δ/Δ^* mice displayed a reduced number of platform crossings (Fig. 3*J*), indicating impaired spatial memory. All groups of mice explored the target quadrant significantly more than other quadrants during the probe trial, indicating that spatial memory had been acquired, but homozygous females showed significantly reduced exploration time in the target quadrant compared with WT females (Fig. 3*K*), consistent with a deficit in spatial learning. There was no effect on swim speed during the MWM test, excluding a reduction in the ability of mice to explore the water maze as a potential explanation for the reduced performance of the mutants (Fig. 3*L*). No significant differences were observed between genotypes in the visible platform task in male mice, excluding poor vision, altered motivation, or sensorimotor alterations as a cause for the deficit in mutant male mice. However, an increased latency to find the visible platform was observed in females (Fig. 3*M*), which might be indicative of visual or motivation changes, or subtle anxiety differences that could be contributing to the deficits observed during the training and testing sessions. Grip strength analysis revealed reduced front- and hindlimb strength in mutant mice (Fig. 3*N,O*). Finally, we performed the contextual fear-conditioning test to assess associative long-term memory. During the training session, both groups of mice increased their freezing behavior following three unconditioned stimuli (US) of 0.7 mA, 2 s foot shocks (Fig. 3*P*). However, the freezing behavior was significantly reduced in *Kdm5b^Δ/Δ^* mice compared with their WT littermates. In the testing session 24 h later, reduced freezing was observed in the mutant animals (Fig. 3*Q*). The increased movement during the training session was also observed when tracking the animals’ movement, but their mean speed was similar during the shock administration, excluding differences in the ability of mutants to discern or respond to the shock (Fig. 3*R*). Nevertheless, the hyperactive phenotype in these mice (Fig. 3*B*) should be considered as an inevitable confounding factor on this test. In summary, although the hyperactivity of the mutants complicates the interpretation of the contextual fear-conditioning test, and confounds exist for the MWM, *Kdm5b^Δ/Δ^* mice exhibited clear deficits in the hippocampus-dependent object location task, leading us to conclude that these mice exhibit hippocampus-dependent learning and memory deficits.

### Altered baseline and learning-induced hippocampal gene expression in *Kdm5b^Δ/Δ^* mice

As KDM5B regulates gene transcription, we next asked if reduced KDM5B demethylase activity affected the transcriptional landscape in the mouse hippocampus. We performed RNA sequencing of adult WT and homozygous hippocampi. We detected 20 DEGs, of which 14 were up- and 6 were downregulated (Fig. 4*A,B* and Extended Data Table 4-1). Intriguingly, two key early response genes associated with learning and memory, *Egr1* and *Npas4*, were among the downregulated genes (Fig. 4*B*). To validate this finding, we quantified transcripts for several immediate early genes (*Egr1*, *Npas4*, *cFos*, *Egr2*, and *Arc*) by quantitative PCRs (qRT-PCR) from a separate set of hippocampal samples. Consistent with the RNAseq data, the expression of *Egr1*, *Npas4*, and *cFos* was significantly downregulated in the mutants (Fig. 4*C*). We next asked if these results translated into alterations in protein levels. As Npas4 is specifically and selectively induced by neuronal activity in the CA3 and DG regions of the hippocampus (Ramamoorthi et al., 2011), Npas4-positive cells were quantified by immunostaining. The number of Npas4-positive cells was reduced in *Kdm5b^Δ/Δ^* mice compared with their WT littermates (Fig. 4*D,E*).

Activity-dependent transcription factors such as Npas4 are crucial for memory formation (Weng et al., 2018; Sun et al., 2020). Thus, we decided to test if *Kdm5b* deficiency is associated with abnormal activity-dependent gene regulation during hippocampus-dependent learning. We compared the hippocampal transcriptomes of three groups of mice with RNAseq: naive animals and mice that were killed 1 or 3 h following training in the contextual fear-conditioning (CFC) paradigm (Extended Data Fig. 5-1 and Table 5-1). As validation, we were again able to confirm the reduction of immediate early gene expression by qRT-PCR in these samples (Extended Data Fig. 5-1A). To understand the differences in activity-induced transcriptional responses between WT and mutant mice, we clustered the DEGs by *k*-means clustering according to their trajectories (Extended Data Table 5-2). Four different clusters were identified, referred to as Clusters 1–4. Cluster 1 exemplified a typical immediate early gene trajectory of rapid, transient induction at 1 h (Fig. 5*A*). These genes were functionally enriched for transcriptional regulation (Extended Data Fig. 5-1G), consistent with the fact that the majority of these genes encode transcription factors such as c-Fos, Fosb, Egr1, Egr2, and Npas4. In agreement with our previous experiments (Fig. 4), this analysis revealed that Cluster 1 genes were downregulated in naive, control mutant animals compared with WTs (Fig. 5*A*). These genes were rapidly induced in mutants, to a larger extent than in WTs, such that they were expressed at the same level as WT controls at both 1 and 3 h post-CFC (Fig. 5*A*). This observation was confirmed by qRT-PCR for the immediate early genes: *Egr2*, *cFos*, *Egr1*, *Npas4*, and *Arc* (Extended Data Fig. 5-1B–F).

10.1523/JNEUROSCI.1544-23.2024.f5-1 Figure 5-1 Gene expression changes in wildtype and *Kdm5b^Δ/Δ^* mice hippocampus following learning. **A)** Immediate early gene expression was analysed by qRT-PCR, relative to *Hprt*, in the same animal cohort used for the RNAseq studies. Two-way ANOVA genotype effect: **p = 0.0052; n = 4 mice/genotype. **B-F)** Immediate early gene expression in *Kdm5b^Δ/Δ^* mice analysed by qPCR, relative to *Hprt*, in a different cohort of mice at baseline (B), 1, 3 and 6 hours after contextual fear conditioning. Note the reduced expression of genes at baseline in Δ/Δ mice; n = 4 mice/genotype. **G)** Gene ontology analyses for the genes obtained within each cluster (yellow, Molecular function; blue, Biological process). **H-J)** Volcano plots displaying gene expression changes detected by DESeq2 between genotypes in baseline mice (H) and 1  h (I) and 3  h (J) after contextual fear conditioning. Each point represents an individual gene, and all DEGs (FDR < 0.05) are highlighted in red (upregulated) or blue (downregulated). The top 10 differentially expressed genes are labelled in purple. Data in A-F is shown as mean ± SEM. Data in (A) was analysed with Student’s t-test. *p < 0.05. Information refers to Figure 5. Download Figure 5-1, TIF file.

10.1523/JNEUROSCI.1544-23.2024.t5-1 Table 5-1 Extended RNA-sequencing data to study transcriptional changes in wild-type and *Kdm5b^Δ/Δ^* mice upon learning as in Figure 5. Mice were trained in the fear conditioning chamber and culled 1  h or 3  h later. Homecage, test-naive mice were used as controls. Dorsal hippocampus was dissected and RNAseq analyses were performed. n = 3 males and 3 females per genotype and timepoint. WT, *Kdm5b^+/+^* mice; HOM, *Kdm5b^Δ/Δ^* mice. Timepoints: 0  h, homecage; 1  h post-fear conditioning and 3  h post-fear conditioning. Download Table 5-1, XLSX file.

10.1523/JNEUROSCI.1544-23.2024.t5-1 Table 5-2 Extended data associated with the clustering of differentially expressed genes in wild-type and *Kdm5b^Δ/Δ^* mice upon learning as in Figure 5. Differentially expressed genes were clustered by their expression trajectories with k means clustering (k = 4). Tables include the list of genes within each cluster. Gene ontology analyses were conducted for each cluster using g:Profiler (https://biit.cs.ut.ee/gprofiler/gost). Download Table 5-2, XLSX file.

We detected three other clusters with different trajectories (Fig. 5*B–D*). Cluster 2 genes showed an induction that gradually increased over time after the learning stimulus (Fig. 5*B*). These genes included late response genes, some with known functions in neurogenesis and synaptic plasticity, for example, *Fgfr1* (Zhao et al., 2007). Similar to Cluster 1, Cluster 2 genes were downregulated in mutants at baseline and reached similar expression levels to WT controls by 3 h (Fig. 5*B*). Cluster 3 included genes that were constitutively overexpressed in homozygous mutants (Fig. 5*C*). This cluster was enriched for transcriptional regulation and cation-binding proteins and included genes encoding transcription factors such as Stat4 and Stat6, as well as Zn finger proteins. These genes included *Cacna1i*, a gene encoding a calcium channel subunit. Gain-of-function mutations in this gene were associated with neurodevelopmental disorders and epilepsy (El Ghaleb et al., 2021). Cluster 3 also included *C1ql2* and *C1ql3*, encoding complement proteins strongly expressed in mossy fibers with a crucial role in the control of synapse stability and number (Matsuda et al., 2016). Cluster 4 genes represented genes that were downregulated in response to a learning stimulus in both WT and mutant hippocampi, with the downregulation more pronounced in mutants (Fig. 5*D*). This cluster was enriched for transcriptional and signaling regulators (Extended Data Fig. 5-1G). Interestingly, these included the *Pde10a* gene, known to enhance fear memories in certain contexts (Guo et al., 2016), but also genes such as *Hgf* known to promote learning and memory, perhaps providing an explanation of learning deficits in our *Kdm5b* mutant mice (Kato et al., 2012). Cluster 4 also includes *Top1*, whose inhibition leads to reduced transcription of genes associated with ASD and neurotransmission regulation (Mabb et al., 2014), and *Arid1b*, which is also implicated in ASD and ID (Moffat et al., 2022).

To better understand how gene expression varied between genotypes at different timepoints, we visualized genes differentially expressed between WT and mutant samples at baseline (home cage test-naive, 0 h), 1 and 3 h after fear-conditioning stimulus. Naive mice showed a small number of DEGs between genotypes (Fig. 5*E*; 12 DEGs). More DEGs were observed between genotypes in activity-induced transcriptional responses 1 h (Fig. 5*F*; 106 DEGs) and 3 h (Fig. 5*G*; 32 DEGs) after training. The majority of DEGs in all conditions were upregulated in the mutant animals, in both female and male mice (Fig. 5*E–G*), consistent with KDM5B acting as a transcriptional repressor.

To understand how the activity-induced transcriptional responses differed between WT and mutants, we compared the hippocampal transcriptomes of home cage control mice with mice 1 and 3 h post-US. WT animals showed the expected increase in the expression of activity-dependent immediate early genes such as *Fosb*, *Npas4*, and *Egr3* 1 h after training (Fig. 5*H*; 1 h). Three hours after training, the gene expression levels of activity genes were back to baseline (Fig. 5*H*; 3 h), consistent with previous studies (Deliu et al., 2018). In addition to the immediate early genes observed in WT mice, *Kdm5b^Δ/Δ^* mice displayed a marked upregulation of many more genes (Fig. 5*I*; 1 h; 252 DEGs). Strikingly, 3 h after training, >200 genes were still differentially expressed, including immediate early genes such as *Fosb*, *Nr4a2*, and *Egr3* (Fig. 5*I*; 3 h; 240 DEGs and Extended Data Fig. 5-1H-J). Together, these data shows that reduced KDM5B demethylase activity alters the transcriptional landscape in the hippocampus in a number of different ways. These include downregulated baseline levels of immediate early genes and more pronounced and sustained activity-dependent upregulation following learning relative to these baseline levels. In conclusion, the abnormal expression of several classes of genes implicated in learning and memory in the hippocampus are likely to contribute to the hippocampus-dependent learning deficits in these mice.

### KDM5B directly regulates learning and memory in the adult hippocampus

Our data thus far suggested that KDM5B has a direct role in learning and memory. However, as KDM5B is known to play important roles during development (Catchpole et al., 2011; Albert et al., 2013), the phenotypes observed in *Kdm5b^Δ/Δ^* mice and other *Kdm5b*-deficient mouse models (Martin et al., 2018; C. Y. Chen et al., 2023) might all be secondary to abnormal brain development and maturation. To determine if KDM5B has a direct role in learning and memory in the adult brain, we knocked down *Kdm5b* expression in the dorsal hippocampus (CA1) of adult mice by AAV-mediated delivery of a *Kdm5b*-specific shRNA (Fig. 6*A,B*).

A quantification of *Kdm5b* mRNA over time by qRT-PCR showed a significant knockdown of *Kdm5b* by 3 weeks after viral delivery and beginning to return to normal levels by 7 weeks (Fig. 6*C*). Importantly, we observed a shKDM5B-dependent downregulation of immediate early genes in naive animals compared with controls that received AAV with a scrambled shRNA (Fig. 6*D*). This observation suggests that the reduced expression of these immediate early genes in *Kdm5b^Δ/Δ^* mice (Fig. 4) is not secondary to developmental abnormalities. *Kdm5b* knockdown (shRNA) mice showed a significant hyperactivity phenotype in the open field versus controls (Fig. 6*E*), recapitulating observations with *Kdm5b^Δ/Δ^* mice (Fig. 3*B*). Importantly, *Kdm5b* knockdown resulted in a substantial deficit in hippocampus-dependent learning and memory in the OLM task (Fig. 6*F*), similar to those in *Kdm5b^Δ/Δ^* mice, indicating that *Kdm5b* is essential for normal long-term memory consolidation in the hippocampus. Exploration time did not show differences between groups in neither training nor testing sessions (Student's *t* test: *p* = 0.4940 for training and *p* = 0.1242 for testing). Furthermore, the shRNA mice exhibited a striking incidence of spontaneous seizures (Fig. 6*G*), suggesting that *Kdm5b* knockdown altered neuronal activation and/or circuitry in the hippocampus.

### Kdm5b regulates synaptic plasticity

To determine if *Kdm5b* knockdown affected synaptic plasticity, we examined short-term potentiation and LTP in acute hippocampal slices from these mice. We measured LTP in the dorsal CA1b region of the hippocampus, which we have found to be critically important for the hippocampus-dependent OLM task in previous studies (Barrett et al., 2011; Vogel-Ciernia et al., 2013). TBS produced an immediate increase in potentiation in slices from control mice that gradually decayed to a plateau level that was ∼50% above pre-TBS baseline (Fig. 6*H*). Hippocampal slices prepared from *Kdm5b* shRNA mice had similar short-term potentiation to control slices; however, the LTP was significantly less stable as the potentiation fell below control levels 1 h post-TBS. The mean potentiation for the last 10 min of 1 h post-TBS was 31 ± 11% for *Kdm5b* shRNA slices and 47 ± 6% for control slices (Fig. 6*I*), suggesting that *Kdm5b* is necessary for the consolidation of LTP in area CA1 of the hippocampus.

We tested whether synaptic events leading to induction of LTP are negatively affected by *Kdm5b* knockdown by measuring short-term plasticity changes including input/output (i/o) curves and paired-pulse facilitation. The i/o curves were comparable with controls and *Kdm5b* shRNA mice (Fig. 6*J*). The slope of the i/o curves was not significantly different between groups (*p* < 0.90; data not shown). Paired-pulse facilitation (Fig. 6*K*), a measure of transmitter release kinetics, also did not show any significant difference between groups and stimulus intervals [two-way ANOVA; *F*_(2,28) _= 2.28; *p* < 0.12]. Thus, it appears that the lack of stability in LTP upon *Kdm5b* knockdown involves cellular events that are set in motion after induction and expression of LTP, an interpretation that is consistent with the observation that short-term potentiation was unaffected in both groups of mice.

## Discussion

Rare coding variants in *KDM5B* are associated with cognitive function in adults, and *KDM5B* is associated with a recessive ID disorder (Faundes et al., 2018; Martin et al., 2018; C. Y. Chen et al., 2023). In this manuscript, we show that mice homozygous for a hypomorphic allele of *Kdm5b* that lacks H3K4me3 demethylase activity exhibited learning and memory deficits. *Kdm5b* deficiency resulted in alterations in the expression of genes implicated in learning and memory. Acute knockdown experiments showed that *Kdm5b* is critical for the normal function of the hippocampus after development has been completed. Notably, two individuals carrying *KDM5B* variants show epileptic spasms and/or generalized seizures (Martin et al., 2018; Mangano et al., 2022), although the latter is more likely associated with a deficit in Nav1. Following *Kdm5b* knockdown in the dorsal hippocampus of adult mice, mice exhibited seizures, consistent with an essential role for *Kdm5b* in maintaining normal circuit homeostasis. These mice had deficits in hippocampus-dependent learning and memory and LTP. Together, this study provides a significant advance in our understanding of the role of KDM5B in learning and memory. Our findings show that KDM5B functions in the adult hippocampus to control learning and memory and imply that at least some of the cognitive deficits associated with KDM5B deficiency could be amenable to treatment in adults.

### Learning and memory genes regulated by KDM5B

Our gene expression analyses revealed several potential ways in which KDM5B deficiency might disrupt memory and learning. First, the expression of activity-dependent, immediate early genes was reduced in *Kdm5b^Δ/Δ^* mice. The deletion of several of these genes has been shown to affect long-term memory formation while leaving short-term memory intact (M. W. Jones et al., 2001; Fleischmann et al., 2003; Ramamoorthi et al., 2011), similar to our findings with *Kdm5b^Δ/Δ^* mice. Second, we found that these immediate early genes were induced by neuronal activation in the hippocampus of *Kdm5b* mutants up to levels equivalent to those in WT mice and therefore hyperactivated relative to baseline levels. Furthermore, these genes were still expressed 3 h after neuronal activation in the mutants, at a timepoint when their expression has returned to baseline in WT mice. This inability of gene expression to return to baseline levels is consistent with the H3K4me3 demethylase function of KDM5B. One might speculate that this reduced expression at baseline and apparent overactivation and altered dynamics of IEGs might disrupt normal memory allocation and consolidation mechanisms in *Kdm5b* mutants, theories that need to be tested for *Kdm5b* and other related disorders (Han et al., 2007, 2008). For instance, Deliu et al. (2018) reported altered trajectories of gene expression in response to conditioning in *Setd5* mutant mice, albeit that baseline gene expression was not markedly different in these mutants compared with WT controls. Third, *Kdm5b* mutant animals also showed an abnormal overexpression of a cluster of genes that was not affected by neural activity. This cluster, which included proteins with a regulatory role over synaptic function and transcription regulation factors, includes those genes constitutively affected by the lack of KDM5B demethylase activity. Our results also implicate KDM5B in the regulation of gene transcription through a number of zinc finger proteins, for example, Zfhx2, Znfx1, Zbtb22, and Zfp810, whose expression is either constitutively affected or abnormally downregulated upon learning in mutant mice (Komine et al., 2012).

Our GO terms analyses also revealed that WT1 is one of the top transcription factors modulating the expression of genes that are downregulated upon a learning stimulus (Cluster 4; Fig. 5*D*). A recent study has showed that WT1 regulates neuronal excitability, LTP, and long-term memory formation. WT1 limits memory strength, thus enabling the memory flexibility required for normal memory formation (Mariottini et al., 2019). The possible relationship between KDM5B and WT1 warrants further investigation.

Together, these data showed that the expression of several different types and classes of genes that are regulated by neuronal activation and necessary for learning and memory is affected by *Kdm5b* deficiency.

### H3K4me3 dysregulation in learning and memory

The finding that mice with *Kdm5b* knockdown in the adult hippocampus exhibit long-term memory deficits suggests that KDM5B has a direct role in learning and memory, independent from any developmental functions. The most likely mechanism is that a deficiency in KDM5B demethylase activity impacts H3K4me3 dynamics and transcriptional output of key learning-associated genes (Gupta et al., 2010; Collins et al., 2019a,b). Abnormalities may be present in different brain regions and cell types and during distinct stages of memory formation, such as allocation (Han et al., 2007, 2008), consolidation (Gupta et al., 2010), and retrieval (Webb et al., 2017). Thus, future studies may need to assess H3K4me3 dynamics at specific gene promoters in different cell types. The conditional deletion of *Kmt2a* and *Kmt2b* in postmitotic neurons using CamKIIcre was sufficient to cause hippocampus-dependent learning and memory deficits, suggesting a direct role for these genes in excitatory neurons (Kerimoglu et al., 2017). Together, these findings further support the notion that H3K4me3 regulation is important for learning and memory and suggest that dysregulation of H3K4me3, in either direction, can lead to memory deficits. However, as chromatin regulators typically function in large, multimolecular complexes, other mechanistic explanations cannot be ruled out. Furthermore, a direct comparison between genes regulated by *Kmt2a* and *Kmt2b* has shown that these factors regulate different genes that likely impact neuronal plasticity in distinct ways (Kerimoglu et al., 2017). The two other H3K4me3-specific demethylases, KDM5A and KDM5C, have also been implicated in learning and memory. A *Kdm5a^−/−^* mouse model has shown to exhibit deficits in the MWM, and *Kdm5c^−/y^* mice have deficits in contextual fear memory and novel object recognition (El Hayek et al., 2020; Vallianatos et al., 2020). Aguilar-Valles et al. (2014) have shown that *Kdm5c* knockdown in the nucleus accumbens in adult mice results in increased H3K4me3 at gene promoters and deficits in methamphetamine-associated memory formation. To the best of our knowledge, *Kdm5a* has not yet been deleted or knocked down in postmitotic neurons in the adult brain, so direct functions for this factor in learning and memory still need to be formally proven. Intriguingly, the contextual fear memory deficits in *Kmt2a^+/−^* and *Kdm5c^−/y^* mice were rescued in *Kmt2a^+/−^*; *Kdm5c^−/y^* double heterozygous mice, suggesting that these factors regulated the same or similar processes, but in opposite directions (Vallianatos et al., 2020).

### Cellular/synaptic functions of KDM5B

Mouse models for other H3K4me regulators such as *Kmt2a*, *Kdm5c*, *Kdm5a*, or *Set1a* display both memory deficits and a decrease in spine density in the hippocampus or cortex (Mukai et al., 2019; El Hayek et al., 2020; Vallianatos et al., 2020). We did not detect significant changes in spine number in the hippocampal stratum oriens and radiatum, or stratum moleculare in the *Kdm5b* mutant mice, suggesting that overt changes in excitatory spine density might not account for the memory deficits in these mutants. The LTP deficit appears to be due to postsynaptic abnormalities, because we did not find any alternations in presynaptic transmitter release. Future studies will need to examine postsynaptic membrane proteins that are known to be involved in LTP and their potential regulation by KDM5B-dependent mechanisms. The exact cell types affected by *Kdm5b* deficiency will also need to be determined.

We recognize certain limitations in our study. We set out to test if KDM5B demethylase activity has a role in long-term memory consolidation. Although our results are consistent with this hypothesis, KDM5B protein levels were also downregulated in *Kdm5b^Δ/Δ^* mice, limiting our ability to conclusively ascertain the contribution of demethylase deficiency versus other KDM5B functions. However, it is also intriguing to note that mutations affecting the demethylase activity of *Drosophila* *kdm5* leads to memory deficits and decreases neurotransmission (Zamurrad et al., 2018; Belalcazar et al., 2021).

The relevance of the increased seizures in *Kdm5b* knockdown mice to clinical phenotypes associated with KDM5B deficiency is still unclear. Chen et al. recently has reported protein-truncating variants of *KDM5B* in individuals with epilepsy, but so far, the clinical description of the small number of individuals with recessive *KDM5B* ID syndrome has not revealed a significant association (Faundes et al., 2018; Martin et al., 2018). We have not observed an obvious increase in spontaneous seizures in our homozygous mouse model. One possibility is that KDM5B deficiency throughout development allows for sufficient homeostatic compensatory mechanisms to ameliorate an overt excitatory/inhibitory imbalance that might lead to spontaneous seizures in some individuals.

Mutations in chromatin-modifying and chromatin-remodeling factors represent a significant proportion of mutations associated with neurodevelopmental disorders and ID (Valencia et al., 2023). The functions of most of these factors in the developing and adult brain are still unknown. As these factors are pleiotropic and likely regulate multiple developmental processes and functions in the brain, identifying their salient functions and mechanisms of action remains a significant challenge. This study, together with other recent reports on other chromatin-remodeling factors (Kerimoglu et al., 2013, 2017; Vogel-Ciernia et al., 2013; C. Y. Chen et al., 2023), suggests that these factors play central roles in the regulation of genes necessary for learning and memory and that ID disorders and cognitive deficits caused by mutation of these factors may to some extent be caused by direct roles in postmitotic neurons. The reduced function of these factors appears to be associated with altered trajectories of activity-dependent gene expression and abnormal engagement of downstream synaptic plasticity mechanisms (Deliu et al., 2018). Understanding these pathological mechanisms may lead to the development of targeted and improved therapies for intellectual disability disorders.

## Ethics Statement

All animal works were approved by local ethical review panels (AWERB, King's College London) and IACUC (UCI 20-095) and work conducted in the UK approved by a Home Office Project license (PP6246123).

## Data Availability Statement

RNAseq data (fastq files) were deposited at the Gene Expression Omnibus archive under the accession number GSE240887 and made freely available upon publication.
