# Supplementary figures and images for: The Intellectual Disability Risk Gene Kdm5b Regulates Long-Term Memory Consolidation in the Hippocampus
Source: J Neurosci. 2024 Apr 4;44(19):e1544232024. doi: 10.1523/JNEUROSCI.1544-23.2024 (PMC11079963; doi:10.1523/JNEUROSCI.1544-23.2024)

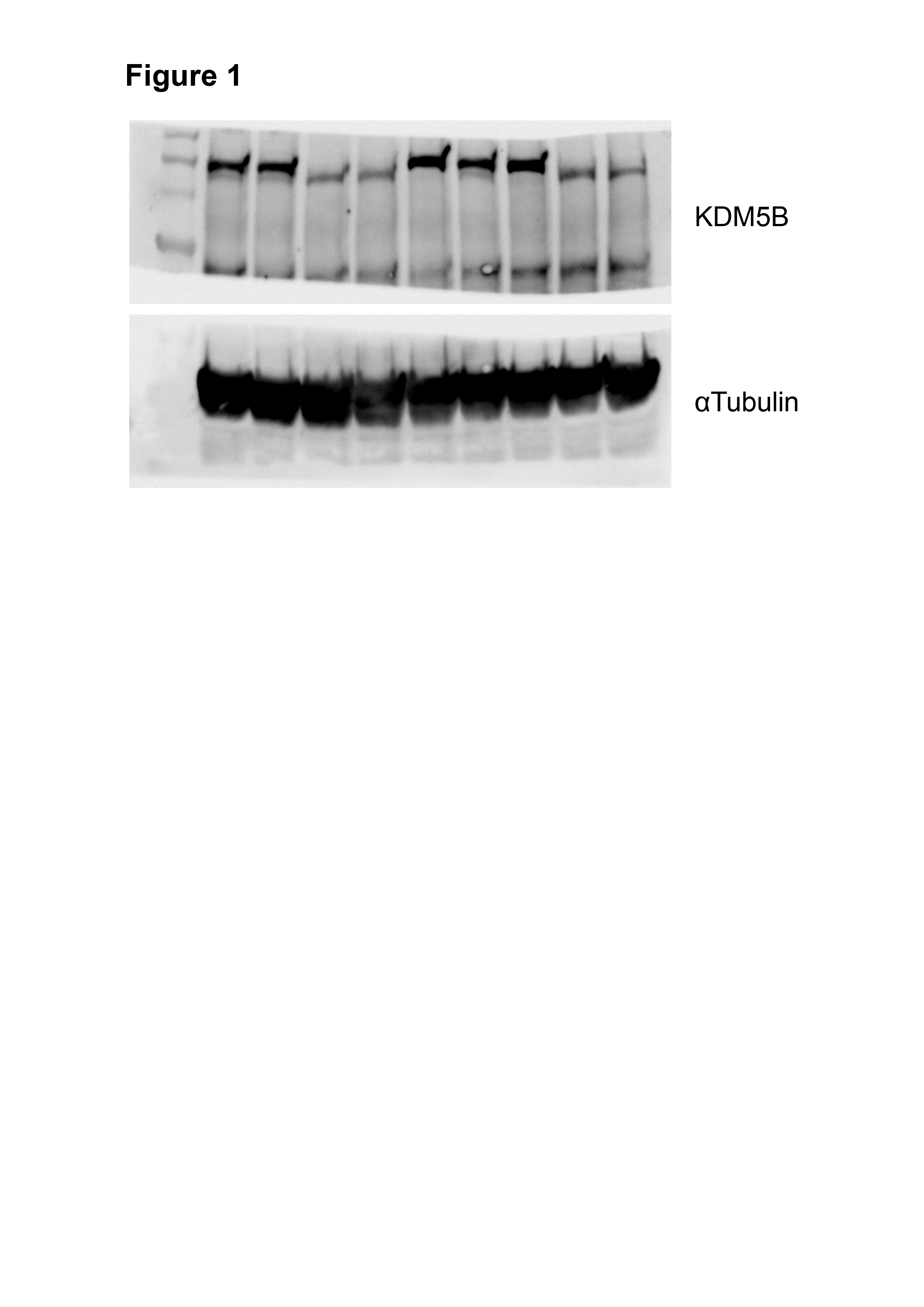

Supplement: Figure 1-1 — Uncropped western blot full scans for the corresponding cropped blots on Figure 1B. Download Figure 1-1, TIF file. [file jneuro-44-e1544232024-s001.tif]

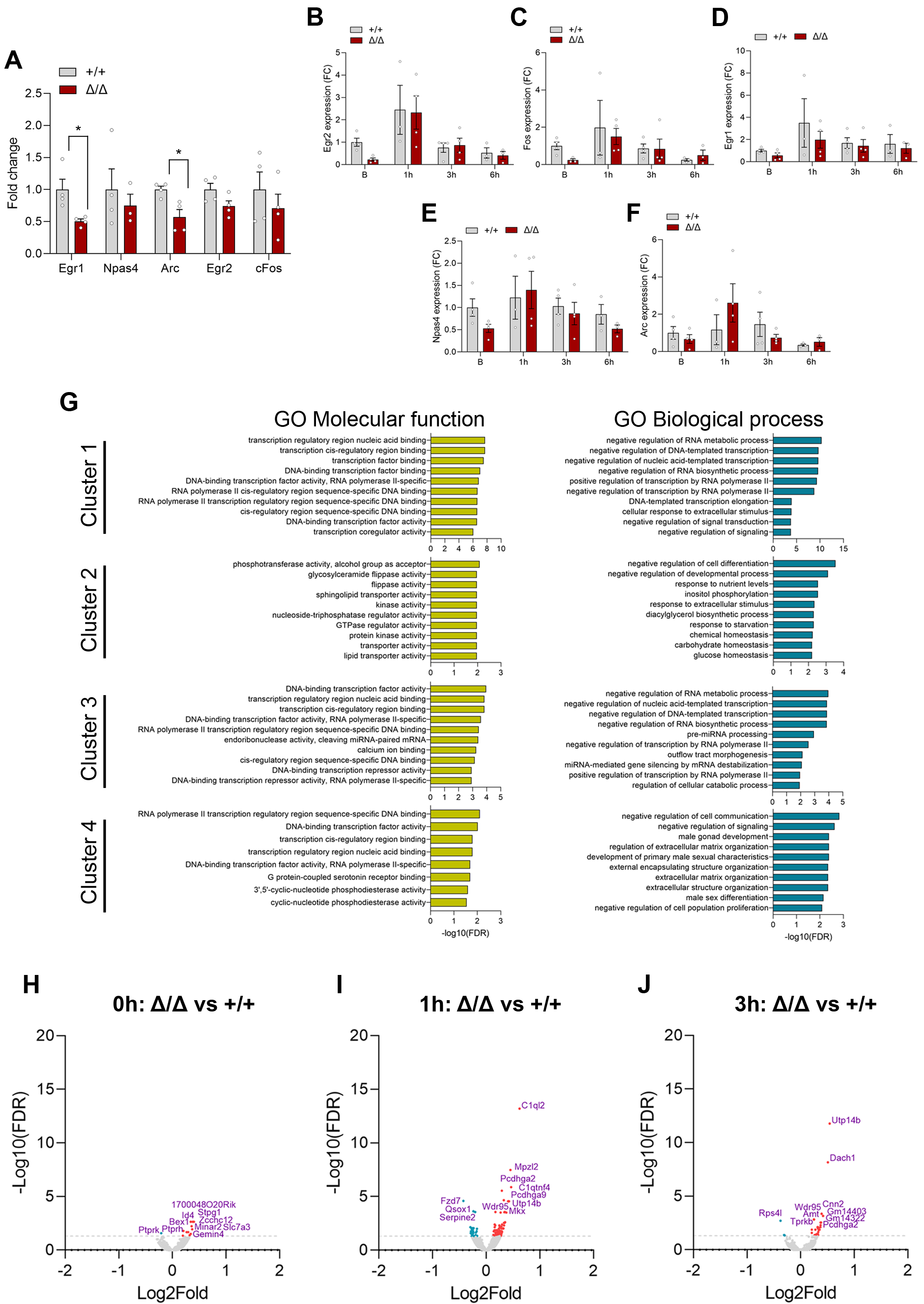

Supplement: Figure 5-1 — Gene expression changes in wildtype and Kdm5bΔ/Δ mice hippocampus following learning. A) Immediate early gene expression was analysed by qRT-PCR, relative to Hprt, in the same animal cohort used for the RNAseq studies. Two-way ANOVA genotype effect: **p = 0.0052; n = 4 mice/genotype. B-F) Immediate early gene expression in Kdm5bΔ/Δ mice analysed by qPCR, relative to Hprt, in a different cohort of mice at baseline (B), 1, 3 and 6 hours after contextual fear conditioning. Note the reduced expression of genes at baseline in Δ/Δ mice; n = 4 mice/genotype. G) Gene ontology analyses for the genes obtained within each cluster (yellow, Molecular function; blue, Biological process). H-J) Volcano plots displaying gene expression changes detected by DESeq2 between genotypes in baseline mice (H) and 1 h (I) and 3 h (J) after contextual fear conditioning. Each point represents an individual gene, and all DEGs (FDR < 0.05) are highlighted in red (upregulated) or blue (downregulated). The top 10 differentially expressed genes are labelled in purple. Data in A-F is shown as mean ± SEM. Data in (A) was analysed with Student’s t-test. *p < 0.05. Information refers to Figure 5. Download Figure 5-1, TIF file. [file jneuro-44-e1544232024-s002.tif]
